# The value of age of onset and family history as predictors of molecular diagnosis in a Swedish cohort of inherited retinal disease

**DOI:** 10.1111/aos.16804

**Published:** 2024-12-06

**Authors:** Karl De Geer, Stefan Löfgren, Anna Lindstrand, Malin Kvarnung, Erik Björck

**Affiliations:** ^1^ Department of Molecular Medicine and Surgery Karolinska Institutet Stockholm Sweden; ^2^ Department of Clinical Genetics and Genomics Karolinska University Hospital Stockholm Sweden; ^3^ Department of Clinical Neuroscience, Division of Ophthalmology and Vision Karolinska Institutet Stockholm Sweden; ^4^ St Erik Eye Hospital Stockholm Sweden

**Keywords:** age of onset, eye diseases, genomics, retinal dystrophies

## Abstract

**Purpose:**

This study aimed to characterize clinical and genetic findings in a Swedish cohort with inherited retinal disease (IRD), identify predictors for achieving a molecular diagnosis and evaluate the effects of increased genetic testing over time.

**Methods:**

Clinical and genetic data from 324 nonrelated IRD index individuals referred for genetic testing in the Stockholm region between 2016 and 2023 were collected retrospectively and analysed by clinical subtype, age of onset and testing period (2016–2020 vs. 2021–2023). Logistic regression was used to calculate odds ratios for age of onset and family history on the likelihood of achieving a molecular diagnosis.

**Results:**

The diagnostic yield was 55% and involved 56 genes. In 10% of solved individuals, the molecular diagnosis refined the clinical diagnosis. For each 1‐year increase in age of onset, the odds of achieving a molecular diagnosis decreased by 3% (odds ratio 0.97, 95% confidence interval 0.96–0.98). A positive family history doubled the odds (odds ratio 2.1, 95% confidence interval 1.3–3.4). The use of genetic testing increased 2.1‐fold and the number of molecular diagnoses increased 1.6‐fold relative to the population of the Stockholm region between the two testing periods.

**Conclusion:**

This study adds to the knowledge of the clinical and genetic landscape of IRDs in Sweden and establishes age of onset and family history as significant predictors for achieving a molecular diagnosis. Increased genetic testing on a population level substantially increased the number of individuals receiving a molecular diagnosis with a high diagnostic yield compared to other rare diseases.

## 1 INTRODUCTION

Inherited retinal diseases (IRDs) constitute a heterogeneous group of more than 90 nonsyndromic and syndromic clinical subtypes characterized by stationary dysfunction or progressive degeneration of the photoreceptors and/or bipolar cells of the outer and inner retinal layers (Schneider et al., 2022; Tatour & Ben‐Yosef, 2020). The prevalence is between 1 in 2000 and 4000 individuals, making IRDs a leading cause of blindness among working‐age adults in the developed world (Heath Jeffery et al., 2021; Liew et al., 2014). IRDs are caused by variants in nuclear or mitochondrial genes and all major inheritance patterns have been described, as well as rare digenic inheritance and disease burden modification by trans‐acting secondary variants (Carss et al., 2017; Kousi et al., 2020). To date, 307 nuclear and mitochondrial genes have been associated with IRDs (Retnet, 2024).

Accurately diagnosing IRDs requires concordance between the clinical subtype and the molecular diagnosis. The molecular diagnosis can rarely be predicted from the phenotype due to significant overlap between clinical subtypes (Carss et al., 2017; Lam et al., 2021). Additionally, interpretation of the molecular diagnosis requires a detailed phenotype because variants in the same gene can cause different clinical subtypes. This is exemplified by the *ABCA4* gene which is responsible for 25% of IRD cases and is associated with conditions such as Stargardt disease, retinitis pigmentosa and cone‐rod dystrophy (Cremers et al., 2020). Adding to the complexity, a single clinical subtype can be associated with multiple genes. This is illustrated by the most common IRD, retinitis pigmentosa, associated with more than 60 genes (Schneider et al., 2022).

The diagnostic yield in genetic testing of IRDs has been estimated to fall between 52% and 74% (Britten‐Jones et al., 2023). The establishment of a molecular diagnosis informs about the prognosis and inheritance pattern of the disease, enables prenatal diagnostics for reproductive decision‐making and positions the individual to benefit from personalized healthcare such as gene‐based therapies and enrolment in clinical trials (Broadgate et al., 2017). The gene therapy voretigene neparvovec‐rzyl (Luxturna) is approved for treatment of *RPE65*‐associated Leber congenital amaurosis by the US Food and Drug Administration and the European Medicines Association (Maguire et al., 2021). For these reasons, the European Reference Network for Rare Eye Diseases has recommended genetic testing for all individuals with suspected or clinically diagnosed IRDs since 2021 (Black et al., 2021).

Historically, genetic testing in IRDs has been limited. A retrospective analysis in an American university healthcare system found that 16% of individuals diagnosed with an IRD were referred for genetic testing. The infrequent use was attributed to high costs, complexity and unfamiliarity with genetic testing by the examining ophthalmologist (Neiweem et al., 2021). Genomic diagnostics is rapidly developing and is of critical importance in the rare disease field. In the Stockholm region, this has led to the establishment of an academic‐clinical collaboration where highly specialized physicians work closely together with trained clinical molecular geneticists and experts in laboratory medicine, genomics and bioinformatics (Stranneheim et al., 2021). Clinical genome sequencing has been implemented as the first‐line test in the diagnostics of many rare diseases, and since 2021, this includes IRDs.

Genome sequencing enables the analysis of single nucleotide variants (SNVs), small insertions and deletions (INDELs), balanced and unbalanced structural variants (SVs), short tandem repeats and stretches of homozygosity and is not limited to the 1–2% of the human genome that is protein‐coding (Stranneheim et al., 2021). Genome sequencing has been shown to be superior to exome sequencing in diverse groups of Mendelian diseases, such as intellectual disability (Lindstrand et al., 2019), neuromuscular disorders (Ek et al., 2023) and IRDs (Weisschuh et al., 2024). It could be argued that, for economic reasons, it would be more cost‐efficient to use targeted testing such as Sanger sequencing to investigate IRD phenotypes that are relatively specific for one gene, such as vitelliform macular dystrophy, choroideremia and retinoschisis. However, we believe the benefits of a streamlined workflow for all IRDs that can detect large SVs, deep‐intronic variants and phenocopy genes outweigh the cost, especially as the price of genome sequencing continues to decrease. An additional benefit is a growing database of genome sequencing data to improve future diagnostics.

The aims of this study were threefold. First, to characterize the clinical and genetic findings in a Swedish IRD cohort in the Stockholm region. Second, to identify clinical predictors for achieving a molecular diagnosis. Third, to investigate how increased use of genetic testing over time has affected cohort characteristics and diagnostic outcomes.

## 2 MATERIALS AND METHODS

### 2.1 Cohort design

Clinical and genetic information was collected from 324 consecutive nonrelated IRD index individuals referred for genetic testing at Karolinska University Hospital between January 2016 and December 2023 (Supplementary Table 1). Ophthalmological examination was performed at St. Erik Eye Hospital, where the vast majority of IRDs in the Stockholm region are diagnosed. The clinical information that was collected from medical records included age of onset, family history (information of a relative with the same phenotype) and consanguinity (second‐degree cousins or closer).

Individuals were categorized into 13 groups based on the clinical diagnosis: ACHM (achromatopsia), CHM (choroideremia), CRD (cone and cone‐rod dystrophy), CSNB (congenital stationary night blindness), LCA (Leber congenital amaurosis), MD (macular dystrophies, excluding Stargardt disease and vitelliform macular dystrophy), MISC (miscellaneous, rare diagnoses observed two or less times), RP (retinitis pigmentosa), RS (retinoschisis), SIRD (syndromic IRD), STGD (Stargardt disease), UIRD (unspecified IRD, usually because of advanced disease) and VMD (vitelliform macular dystrophy).

The diagnoses in the MISC category were Aland eye disease, Bietti crystalline dystrophy, blue cone monochromacy, Bornholm eye disease, Bothnia retinal dystrophy, bradyopsia, central areolar choroidal dystrophy, Doyne honeycomb retinal dystrophy, enhanced S‐cone syndrome, fundus albipunctatus, retinal dystrophy with nanophthalmos, occult macular dystrophy, drusen maculopathy, retinitis punctata albescens, salt and pepper retinopathy and Sveinsson chorioretinal atrophy.

Individuals with the following clinical diagnoses were excluded from the cohort: albinism, Alport syndrome, autoimmune retinal disease, basal laminar drusen, chronic progressive external ophthalmoplegia, Kearns–Sayre syndrome, Leber hereditary optic neuropathy, optic atrophy and vitreoretinopathy. Additionally, affected relatives of the index individual that had also been investigated were excluded (*n* = 26).

### 2.2 Definition of age of onset from medical records

The age of onset of visual symptoms was not recorded uniformly in the medical records. In 66% (*n* = 214) of individuals, the age of onset was specified to a year or year interval. In 9% (*n* = 30), the age at clinical diagnosis was used to approximate the age of onset. In 5% (*n* = 17), an ophthalmological examination revealed findings suggestive of an IRD in an individual without subjective symptoms, in which case the age at examination was used to approximate the age of onset.

In the remaining 20% of individuals, the age of onset was recorded as a phrase instead of years. The phrases were grouped and converted to an age of onset: ‘Childhood onset’ (10%, *n* = 33): 7 years of age. ‘Past few years’ (6%, *n* = 18): 3 years prior. ‘Symptoms for a long time’ (1%, *n* = 4): 10 years prior. ‘Teens’ (1%, *n* = 4): 15 years of age. ‘Early teens’ (1%, *n* = 3): 13 years of age. ‘Several decades’ (<1%, *n* = 1): 30 years prior.

In the analysis of differences in cohort characteristics by paediatric and adult age of onset, paediatric age of onset was defined as occurring before 18 years of age.

### 2.3 Genetic analysis

Genomic DNA was isolated from whole blood using QIAsymphony (QIAGEN, Hilden, Germany) and the QIAsymphony DSP DNA Midi Kit (cat. no. 937255, QIAGEN, Hilden, Germany) according to the manufacturer's protocol.

Our workflow of clinical genome sequencing has previously been described in detail (Stranneheim et al., 2021) and was implemented as the first‐line test for IRDs in 2021. In short, all cases were mapped to GRCh37 (hg19). An in‐house developed analysis pipeline (MIP; https://github.com/Clinical‐Genomics/MIP) was used to detect SNVs, INDELs and SVs (deletions/duplications and balanced aberrations) in a comprehensive panel of known IRD genes. Variants were ranked and visualized in Scout, an in‐house analysis software (Scout; https://github.com/Clinical‐Genomics/scout) (Stranneheim et al., 2021). Larger SVs (>50 kilobases) were visualized in the Cytosure Interpret Software (Oxford Gene Technology) after conversion with a custom program (vcf2cytosure; https://github.com/NBISweden/vcf2cytosure), as described previously (Lindstrand et al., 2019).

SVs were verified by Sanger sequencing with breakpoint polymerase chain reaction (PCR), Multiplex Ligation‐dependent Probe Amplification or chromosomal microarray using a custom 180 000 oligonucleotide array, designed with an even distribution across the genome, with ∼18 Kb probe spacing (AMADID:031035, Oxford Gene Technology, Begbroke, Oxfordshire, UK). SNVs with low‐quality parameters and INDELS were verified by conventional PCR and Sanger sequencing.

Genome sequencing was used in 166 individuals (51%) of which 163 were analysed as singletons and three as trios. An in‐house IRD gene panel was used in 137 (85%) of the individuals. The number of nuclear and mitochondrial genes in the panel has been updated during the study period and has varied between 325 and 364 genes (Supplementary Table 2). In 20 individuals (11%), a smaller subset of IRD genes were analysed due to a specific clinical request. In seven individuals (4%), there were syndromic symptoms such that a different gene panel was considered more appropriate (intellectual disability, ciliopathy, retinal dystrophy with nanophthalmos).

The DNA samples of the 160 individuals not analysed by genome sequencing were sent to external laboratories for single gene analysis, targeted gene panels or exome sequencing (Asper Biogene: *n* = 2, Blueprint Genetics: *n* = 124, CeGaT: *n* = 18, Centogene: *n* = 12, Fulgent Genetics: *n* = 1 and GeneDx: *n* = 2).

### 2.4 Variant interpretation

SNVs, INDELs and SVs were classified according to the American College of Medical Genetics and Genomics (ACMG)/Association for Molecular Pathology (AMP) guidelines as described previously (Lindstrand et al., 2022; Richards et al., 2015). Class 4 and 5 variants were reported to the referring doctor. Selected class 3 variants, variants of uncertain significance, were reported if they were on the verge of classification as likely pathogenic and were consistent with the phenotype of the individuals or due to compound heterozygosity with a pathogenic variant.

### 2.5 Statistical analysis

The cohort characteristics were summarized using descriptive statistics. Categorical variables were presented with percentages (sex, consanguinity, positive family history, full‐field electroretinography and inheritance patterns). The continuous variable age of onset was presented with the median and range since the data were not normally distributed according to evaluation of histograms and the Shapiro–Wilk normality test. Significance testing was performed using the chi‐squared test for categorical variables and the Wilcoxon rank‐sum test for continuous variables. Differences in age of onset between inheritance patterns were compared with the Kruskal–Wallis test. Pairwise comparisons were conducted using Dunn's post hoc test with Bonferroni correction to account for multiple testing. Statistical significance was defined as *p* < 0.05.

To estimate the odds ratios and the 95% confidence intervals (CI) of predictors for the likelihood of achieving a molecular diagnosis, a univariable and a multivariable logistic regression model were used. In the univariable model, age of onset was the sole predictor. In the multivariable model, the predictors were age of onset, family history status and sex. The goodness of fit of the models was evaluated using the Hosmer–Lemeshow test, with the data divided into 10 groups. The test results were non‐significant for both the univariable and the multivariable models (chi‐squared statistic = 10.9 and 6.2, *p* = 0.21 and *p* = 0.62), indicating that the models fit the data adequately (Hosmer et al., 2013). Statistical analyses were performed in R statistical software version 4.3.2 (2023‐10‐31) (R Core Team, 2023).

### 2.6 Calculation of genetic testing in relation to the population

The number of genetic tests and the number of individuals diagnosed per year per million inhabitants in the Stockholm region were calculated for two time periods: 2016–2020 and 2021–2023. Individuals with multiple genetic tests during the study period were registered by the year of the first genetic test.

The number of genetic tests and the number of individuals with a molecular diagnosis in each of the two time periods were divided by the average population in the Stockholm region and the number of years in the period. The result was multiplied by one million. The average population in the Stockholm region between 2016 and 2020 was 2 338 080 and the average population between 2020 and 2023 was 2 438 570 (Region Stockholm, 2024).

### 2.7 Ethical approval

Ethical approval was obtained by the Regional Ethical Review Authority in Stockholm (ethics permit number 2023‐03055‐01, addendum 2023‐07837‐02). The permit allowed for analysis of clinical and genetic data on a cohort level. The approval did not require us to obtain written consent. The research followed the principles of the Helsinki Declaration.

## 3 RESULTS

### 3.1 Clinical and genetic characteristics

The cohort included 324 unrelated individuals (176 males and 148 females) referred for genetic testing due to a clinical IRD diagnosis (Table 1). All individuals underwent a comprehensive ophthalmological examination which included best‐corrected visual acuity (99% of individuals), optical coherence tomography (94%), fundus autofluorescence photography (90%), full‐field electroretinography (65%) and visual field testing (65%).

**TABLE 1:** Clinical and genetic characterization of the cohort by clinical subgroup.

Category	Subcategory	Overall	RP	CRD	STGD	SIRD	VMD	UIRD	MISC	MD	RS	CSNB	CHM	LCA	ACHM
Individuals (n)		324	89	45	44	34	22	20	19	17	10	9	7	5	3
Sex (%)	Female	46	52	51	59	38	55	40	42	59	0	0	0	40	0
	Male	54	48	49	41	62	45	60	58	41	100	100	100	60	100
Median age of onset (years)		18 (0–84)	22 (2–70)	17 (0–84)	26 (5–79)	14 (0–55)	37 (4–79)	10 (0–70)	14 (0–68)	52 (5–65)	7 (5–21)	7 (0–33)	15 (7–40)	0 (0–1)	0 (0–7)
Consanguinity (%)		14	15	18	14	32	0	10	21	0	0	0	0	20	0
Positive family history (%)		37	44	31	20	35	45	45	37	24	60	22	71	40	33
ff‐ERG performed (n)		211	60	42	19	25	5	12	15	12	2	9	3	4	3
ff‐ERG finding (%)	Normal	10	2	0	47	8	60	0	0	33	50	22	0	0	0
	Cone dysfunction	15	0	19	26	4	40	0	33	58	0	11	0	0	100
	Rod dysfunction	1	0	0	0	4	0	0	0	0	0	11	0	0	0
	Rod‐ and cone dysfunction	73	98	81	26	84	0	100	67	8	50	56	100	100	0
Diagnostic yield (%)		55	54	36	64	47	73	55	53	35	100	44	100	60	67
Inheritance pattern (%)	Autosomal dominant	25	27	50	0	0	100	18	0	100	0	0	0	0	0
	Autosomal recessive	59	63	50	100	100	0	64	90	0	0	25	0	100	100
	X‐linked	16	10	0	0	0	0	18	10	0	100	75	100	0	0

Abbreviations: n, number of individuals; ff‐ERG, full‐field electroretinography; ACHM, Achromatopsia; CHM, Choroideremia; CRD, Cone and cone‐rod dystrophy; CSNB, Congenital stationary night blindness; LCA, Leber congenital amaurosis; MD, Macular dystrophy; MISC, Miscellaneous (2 observations or less); RP, Retinitis pigmentosa; RS, Retinoschisis; SIRD, Syndromic IRD; STGD, Stargardt disease; UIRD, Unspecified IRD; VMD, Vitelliform macular dystrophy.

The most common clinical subgroups were retinitis pigmentosa (27%), cone and cone‐rod dystrophy (14%) and Stargardt disease (14%). The median age of onset was 18 years (range: 0–84 years). The median time from age of onset to genetic testing was 10 years. If ff‐ERG was performed, the result was pathological in 90% of cases. The diagnostic yield was 55%, defined as ACMG class 4 and 5 variants. There was a wide range in diagnostic yield between clinical subgroups, from 35% in macular dystrophy to 100% in choroideremia and retinoschisis. In 15% of the cohort, there were genetic findings suggestive but not sufficient for a molecular diagnosis. Of the suggestive findings, 12% were variants of uncertain significance (VUS) and 3% were heterozygous likely pathogenic or pathogenic variants with no compound variant in a recessive gene consistent with the phenotype.

### 3.2 Genes and disease‐causing variants

A molecular diagnosis was established in 177 individuals, with 282 disease‐causing variants across 56 different genes (Table 2). The most common genes by the number of affected individuals were *ABCA4* (20%), *BEST1* (8%), *RS1* (6%), *USH2A* (6%) and *PRPH2* (5%). Notably, 29 of the 56 genes (52%) were found in only one individual. The clinical subgroup with the greatest genetic heterogeneity was retinitis pigmentosa, with disease‐causing variants detected in 20 different genes.

**TABLE 2:** Distribution of genes with disease‐causing variants by clinical subgroup.

Clinical subgroup	Genes with disease‐causing variants
ACHM	*CNGB3* (1); *PDE6H* (1)
CHM	*CHM* (7)
CRD	*ABCA4* (7); *CRX* (3); *PROM1* (2); *GUCY2D* (1); *KCNV2* (1); *PRPH2* (1); *RHO* (1)
CSNB	*CACNA1F* (3); *RLBP1* (1)
LCA	*CRB1* (1); *NMNAT1* (1); *RPGRIP1* (1)
MD	*PRPH2* (4); *GUCY2D* (1); *IMPG1* (1)
MISC	*CYP4V2* (2); *RDH5* (2); *RLBP1* (2); *CACNA1F* (1); *MFRP* (1); *NR2E3* (1); *RGS9* (1)
RP	*USH2A* (7); *EYS* (5); *PRPF31* (5); *RHO* (4); *RPGR* (4); *CNGA1* (3); *CRB1* (3); *PCARE* (2); *PDE6A* (2); *RP1* (2); *SNRNP200* (2); *CEP290* (1); *CNGB1* (1); *NR2E3* (1); *PDE6B* (1); *PRPH2* (1); *RP1L1* (1); *RP2* (1); *SLC24A1* (1); *TOPORS* (1)
RS	*RS1* (10)
SIRD	*USH2A* (3); *CWC27* (2); *ADGRV1* (1); *ALMS1* (1); *CDH23* (1); *CEP250* (1); *CEP78* (1); *GNPTG* (1); *LAMA1* (1); *MYO7A* (1); *POMGNT1* (1); *RNU4ATAC* (1); *SCLT1* (1)
STGD	*ABCA4* (28)
UIRD	*BEST1* (2); *ABCA4* (1); *ADAM9* (1); *BBS1* (1); *CHM* (1); *CLN3* (1); *CNGB3* (1); *RDH12* (1); *RPE65* (1); *RPGR* (1)
VMD	*BEST1* (12); *PRPH2* (3); *IMPG1* (1)

Abbreviations: ACHM, Achromatopsia; CHM, Choroideremia; CRD, Cone and cone‐rod dystrophy; CSNB, Congenital stationary night blindness; LCA, Leber congenital amaurosis; MD, Macular dystrophy; MISC, Miscellaneous (2 observations or less); RP, Retinitis pigmentosa; RS, Retinoschisis; SIRD, Syndromic IRD; STGD, Stargardt disease; UIRD, Unspecified IRD; VMD, Vitelliform macular dystrophy.

**FIGURE 1:**
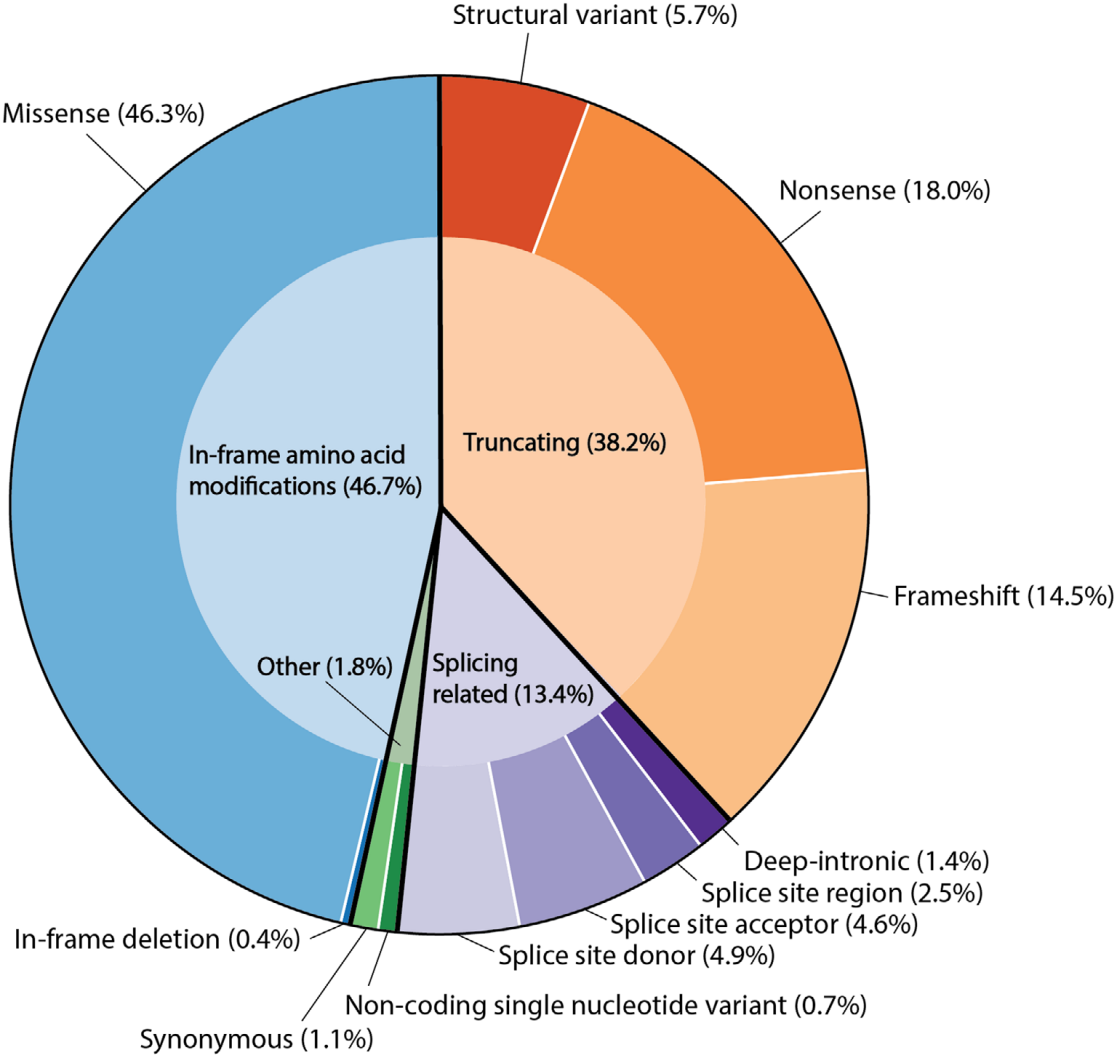
All 282 class 4 and 5 variants in the cohort grouped by variant type.

Class 4 and 5 variants were grouped by type and homozygous variants counted twice (Figure 1). The genes that accounted for the highest proportion of variants were *ABCA4* (26%), *USH2A* (7%) and *BEST1* (5%). SVs were detected in *CHM* (*n* = 4), *CLN3* (*n* = 1), *CRX* (*n* = 1), *CWC27* (*n* = 1), *IMPG1* (*n* = 1), *PRPF31* (*n* = 3), *RGS9* (*n* = 1), *RP2* (*n* = 1), *RPGR* (*n* = 1) and *USH2A* (*n* = 2). One non‐coding SNV was observed in *RNU4ATAC*. Deep‐intronic variants were detected in *ABCA4*, *USH2A* and *NMNAT1*. The variant in *NMNAT1*, c.‐71G > A, was novel, confirmed to affect splicing by RNA‐sequencing and classified as likely pathogenic. Additionally, a novel deep‐intronic VUS was detected in *WDR19*, c.1982 + 963G > A.

### 3.3 Reclassification or specification of clinical subgroup

The clinical subgroup was reclassified or specified by the molecular diagnosis in 10% (*n* = 18) of individuals with a molecular diagnosis. The clinical subgroup was reclassified in an individual with suspected Stargardt disease where the molecular diagnosis was consistent with vitelliform macular dystrophy (*BEST1*), and in an individual with suspected blue cone monochromacy, the molecular diagnosis was consistent with achromatopsia (*CNGB3*). In four individuals with clinical suspicion of a nonsyndromic IRD, the molecular diagnosis was associated with a syndromic IRD, and syndromic features were identified by additional phenotyping (*BBS1*, *CLN3*, *GNPTG*, *CWC27*). Furthermore, in three individuals with clinical suspicion of a specific syndromic IRD, the molecular diagnosis was suggestive of a different syndromic IRD (*ALMS1*, *CEP250* and *RNU4ATAC*). Lastly, in nine individuals, the clinical subgroup was ‘Unspecified IRD’ that was specified by the molecular diagnosis.

### 3.4 Predictors for achieving a molecular diagnosis

Age of onset, family history status and consanguinity were tested as potential predictors for achieving a molecular diagnosis. Age of onset and family history status were significantly different between individuals with and without a molecular diagnosis. A trend towards significance was observed for consanguinity (Table 3).

**TABLE 3:** The association between clinical variables and the likelihood of achieving a molecular diagnosis.

Characteristic	Diagnosis, *n* = 177	No diagnosis, *n* = 147	*p*‐value
Consanguinity (%)	18	10	0.056
Median age of onset (years)	14 (0–68)	27 (0–84)	–
Positive family history (%)	45	27	0.001

*Note*: Chi‐squared tests were used for categorical variables and Wilcoxon rank‐sum test for age of onset. "–" indicates *p*‐value < 0.00001.

Abbreviations: *n*, number of individuals.

A multivariable logistic regression model was used to estimate the effect sizes of age of onset and family history status on the odds of achieving a molecular diagnosis, with adjustment for sex as a covariate (Table 4). No statistically significant interactions were identified between predictors (all *p*‐values >0.5), and no significant multicollinearity was detected (variance inflation factors all <1.03).

**TABLE 4:** Multivariable logistic regression analysis of predictors for the likelihood of achieving a molecular diagnosis.

Variable	Odds ratio	CI lower	CI upper	*p*‐value
Age of onset	0.97	0.96	0.98	–
Family history	2.06	1.27	3.38	0.004
Sex (male)	0.93	0.58	1.49	0.77

*Note*: *p*‐values refer to the significance of the coefficient estimates. "–" indicates *p*‐value < 0.00001.

Abbreviations: CI, 95% confidence interval.

**FIGURE 2:**
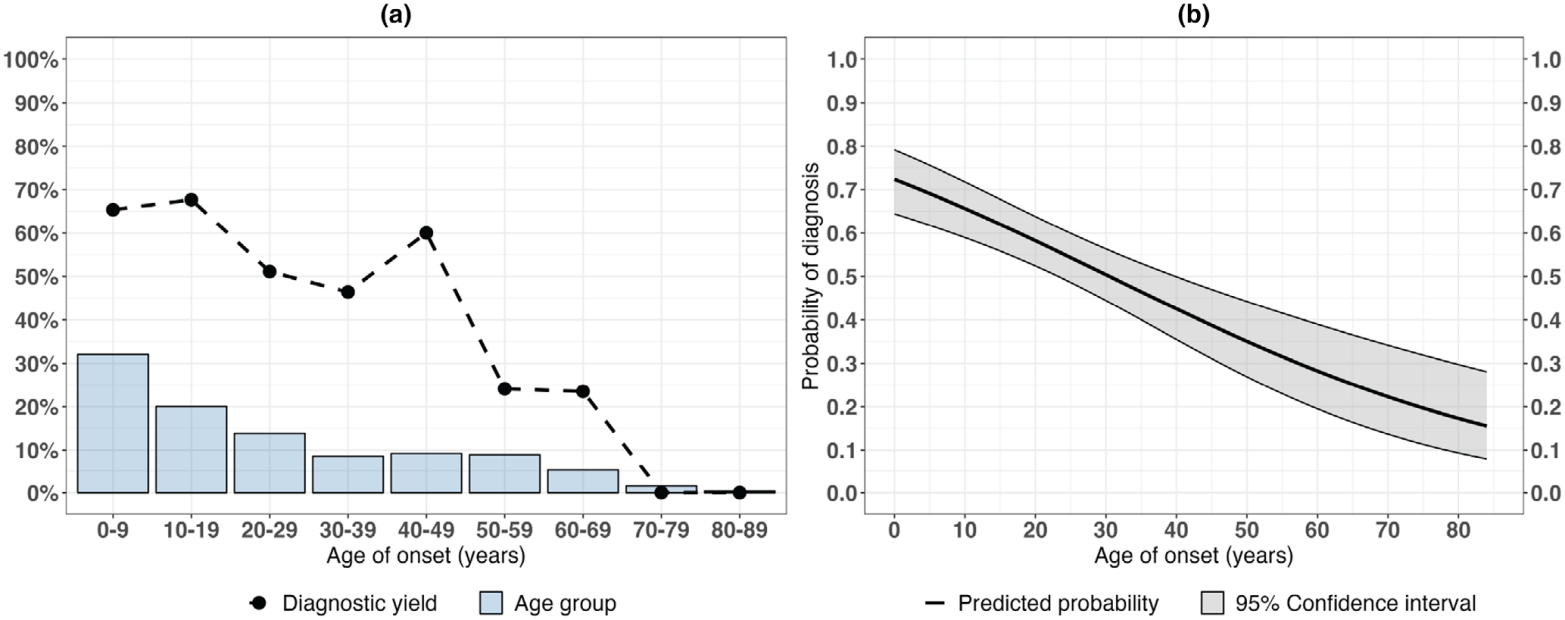
Association of age of onset and the likelihood of achieving a molecular diagnosis. (a) Diagnostic yield in the cohort grouped by age of onset. (b) Predicted probabilities of achieving a molecular diagnosis by age of onset in a univariable logistic regression model.

A positive family history increased the odds for achieving a molecular diagnosis 2.1‐fold, while each additional year in age of onset decreased the odds by 3%. The inverse relationship between age of onset and achieving a molecular diagnosis was further characterized by plotting diagnostic yield across groups of increasing age of onset (Figure 2a) and by visualizing the predicted probabilities for achieving a molecular diagnosis in a univariable logistic regression model (Figure 2b).

### 3.5 Age of onset and associations with clinical and genetic characteristics

**FIGURE 3:**
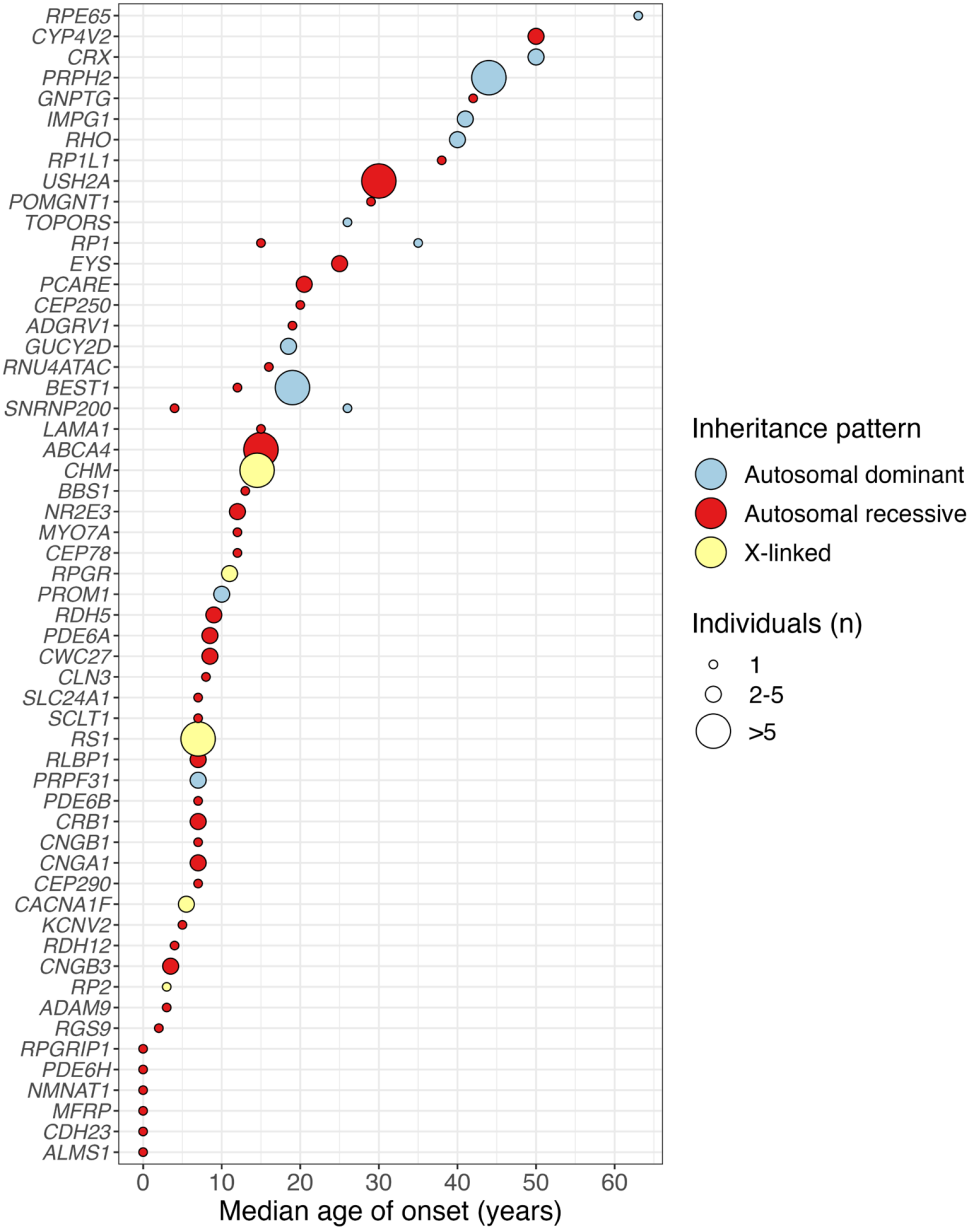
Age of onset and inheritance patterns in the 56 genes with disease‐causing variants.

Age of onset was analysed for each inheritance pattern and for all genes with disease‐causing variants (Figure 3). The median age of onset was 30 years for autosomal dominant inheritance, 13.5 years for autosomal recessive inheritance and 9.5 years for X‐linked inheritance. The 13 genes with the earliest onset were associated with autosomal recessive and X‐linked inheritance, while five of the seven genes with the latest onset were associated with autosomal dominant inheritance. These differences were statistically significant, with autosomal dominant inheritance showing a significantly later onset compared to both autosomal recessive (Z = 3.43, adjusted *p* = 0.002, Kruskal–Wallis test and Dunn's post hoc test with Bonferroni correction) and X‐linked patterns (Z = 3.73, adjusted *p* = 0.0006). No significant difference was observed between autosomal recessive and X‐linked patterns (Z = 1.34, adjusted *p* = 0.543).

Age of onset was further explored for associations to specific clinical subgroups and clinical characteristics. To that end, the cohort was divided into a paediatric group (onset before 18 years of age) and an adult group (onset at 18 years or older) (Table 5). In the paediatric group, the most prevalent clinical subtypes were retinitis pigmentosa (23%), cone and cone‐rod dystrophy (15%), syndromic IRD (13%), Stargardt disease (10%) and unspecified IRD (10%). The most common genes were *ABCA4* (20%), *RS1* (8%), *BEST1* (7%), *CHM* (5%) and *CACNA1F* (4%). In the adult group, the most prevalent clinical subtypes were retinitis pigmentosa (33%), Stargardt disease (18%), cone and cone‐rod dystrophy (13%), vitelliform macular dystrophy (11%) and macular dystrophy (9%). The most common genes were *ABCA4* (21%), *PRPH2* (13%), *USH2A* (13%), *BEST1* (10%) and *CHM* (4%).

**TABLE 5:** Cohort characteristics grouped by paediatric or adult age of onset.

Variable	Subcategory	Adult onset	Paediatric onset	*p*‐value
Total investigations (*n*)		164	160	NA
Sex (%)	Female	50	41	NA
	Male	50	59	NA
Median time to genetic testing (years)		9 (0–55)	12 (−4–69)	0.011
Diagnostic yield (%)		43	66	–
Positive family history (%)		34	41	0.228
Consanguinity (%)		7	21	0.0003
Refined clinical diagnosis (%)		6	13	0.168
Inheritance pattern (%)	X‐linked	7	22	0.016
	Autosomal dominant	41	15	0.0002
	Autosomal recessive	52	63	0.189

*Note*: In one individual genetic testing was performed 4 years prior to the onset of visual symptoms due to suspicion of a syndrome. This is the reason for the negative range in the ‘Median time to genetic testing (years)’. "–" indicates *p*‐value < 0.00001.

Abbreviations: *n*, number of individuals; NA, not applicable.

### 3.6 Genetic testing frequency during the study period

Because the cohort was collected from a distinct geographical region with a known population, the use and outcome of genetic testing could be analysed over time and in relation to the population of the Stockholm region. Between 2016 and 2020, the yearly number of referrals for genetic testing was 20, 14, 29, 38 and 38 (mean 28). The number of yearly referrals between 2021 and 2023 was 59, 63 and 63 (mean 62). To explore how the increased use of genetic testing affected cohort characteristics, the cohort was divided into two groups based on the year of referral: 2016–2020 and 2021–2023 (Table 6).

**TABLE 6:** Comparison of cohort characteristics between time periods.

Variable	2016–2020	2021–2023	*p*‐value
Total investigations (*n*)	139	185	NA
Mean yearly tests per million (*n*)	11.9	25.3	NA
Mean yearly diagnoses per million (*n*)	7.6	12.0	NA
Diagnostic yield (%)	64	48	0.005
Genome sequencing (%)	14	78	–
Positive family history (%)	44	32	0.036
Median age of onset (years)	14 (0–70)	26 (0–84)	–

*Note:* Mean yearly tests and diagnoses per million are in relation to the average population of the Stockholm region for each period. "–" indicates *p*‐value < 0.00001.

Abbreviations: *n*, number of individuals; NA, not applicable.

Relative to the population of the Stockholm region, the number of genetic tests per million inhabitants per year increased 2.1‐fold between the two time periods. While the number of individuals diagnosed per million inhabitants per year increased 1.6‐fold, the diagnostic yield decreased from 64% to 48%. Significant cohort differences were observed in the distribution of the predictors age of onset and family history status between the two time periods. The median age of onset increased by 12 years, and the proportion of individuals with a positive family history decreased by 12%.

## 4 DISCUSSION

In this study, we present the clinical and genetic findings from a Swedish IRD cohort in the Stockholm region. Although several prior studies have characterized IRD cohorts, few have specifically examined clinical predictors associated with achieving a molecular diagnosis. Our findings indicate that age of onset and family history are independent and statistically significant predictors of achieving a molecular diagnosis. The finding lays a foundation for future research into genetic and non‐genetic factors contributing to disease in groups with low diagnostic yields, such as late‐onset IRDs. Furthermore, we assess the impact of increased genetic testing on a population level. The critical role of a molecular diagnosis in the era of precision medicine needs to be balanced against the economic cost of genetic testing. We show that a 2.1‐fold increase in genetic testing led to a 1.6‐fold increase in molecular diagnoses, which we believe supports genetic testing for all individuals with suspected or clinically diagnosed IRD. In the following sections, these topics will be addressed in detail.

First, the clinical subtypes and genetic findings in the cohort were characterized. The most prevalent clinical subtypes were retinitis pigmentosa, cone and cone‐rod dystrophy and Stargardt disease, which is consistent with findings in European cohorts (Carss et al., 2017; Weisschuh et al., 2024). The most common genes with disease‐causing variants were *ABCA4*, *BEST1*, *RS1*, *USH2A* and *PRPH2*. *ABCA4*, *USH2A* and *PRPH2* have been reported among the most prevalent IRD‐associated genes globally (Pontikos et al., 2020). However, *BEST1* and *RS1* appear to be more common in the Nordic countries. A Swedish study on the value of renewed genetic testing found *RS1* among the top five genes, while *BEST1* was common in their previously solved cohort (Areblom et al., 2023). Both genes were among the most common in a Norwegian cohort, and while *BEST1* was among the top five genes in a Danish study, *RS1* was the most common gene in a Finnish paediatric cohort (Avela et al., 2019; Holtan et al., 2020; Jespersgaard et al., 2019).

The distribution of variant types was comparable to the Global Retinal Inherited Disease (GRID) data set, except for SVs (Schneider et al., 2022). In‐frame amino acid modifications constituted 46.3% (GRID: 42.1%), truncating variants 38.2% (GRID: 41.3%), splicing related variants 13.4% (GRID: 15.7%) and other variant types 1.8% (GRID: 0.5%). SVs, a subset of truncating variants, made up 5.7% of our cohort compared to 0.84% in GRID. Schneider et al. attributed this difference to GRID including only large cohorts, while SVs are more often reported in smaller studies. Other estimates suggest SVs account for 7–9% of disease‐causing variants, aligning more closely with our findings (Ellingford et al., 2018; Zampaglione et al., 2020).

Second, we established age of onset and family history status as statistically significant predictors for achieving a molecular diagnosis. Autosomal recessive and X‐linked inheritance patterns were associated with a significantly younger age of onset compared to autosomal dominant inheritance. In our univariable logistic regression model, each additional year in age of onset decreased the odds for achieving a molecular diagnosis by 3%. An individual with age of onset at 10 years had a predicted probability of a molecular diagnosis of 66%, compared to a probability of 35% in an individual with age of onset at 50 years. Previous studies in cohorts with retinitis pigmentosa have observed a decrease in diagnostic yield with increasing age of onset and suggested that non‐genetic factors, such as infectious retinal vasculitis, drug‐induced retinal toxicity or autoimmune retinopathies, may mimic retinitis pigmentosa in individuals with later onset (Gao et al., 2019; Kim et al., 2021; Shanks et al., 2013). However, a possible interpretation of our finding that older age of onset is associated with autosomal dominant inheritance is that unsolved late‐onset IRDs might be caused by thus far undetected low‐penetrance, dominant‐acting variants.

Age of onset was further analysed by grouping the cohort into paediatric and adult onset. We found that genetic testing in individuals with paediatric onset provides benefits beyond an increased diagnostic yield. Genetic testing helps establish an accurate diagnosis, as 78% of those whose clinical diagnosis was reclassified or specified by molecular findings had a paediatric onset. Additionally, genetic testing can identify individuals with syndromic IRDs and enable early and proactive monitoring of multisystem manifestations. Among those with syndromic IRDs, 62% had a paediatric onset. Furthermore, achieving a molecular diagnosis early in the disease course, regardless of age of onset, is essential for identifying candidates for emerging treatments and clinical trials. Gene therapy is available for *RPE65*‐associated autosomal recessive retinal dystrophy, where early treatment may offer the best therapeutic outcomes (Hu et al., 2021). We did not detect any such cases, though one individual carried the autosomal dominant variant *RPE65* c.1430A > G associated with retinitis pigmentosa 87 with choroidal involvement. The age of onset for this individual was 63 years, which is late compared to the commonly reported onset in the fourth of fifth decade (Kiang et al., 2020). Despite the advantages of early genetic testing, we found that individuals with paediatric onset had a longer time from disease onset to genetic testing compared to those with adult onset (9 vs. 12 years). This delay may represent a missed opportunity for early intervention and targeted care.

In the multivariable logistic regression analysis, we found an odds ratio for family history status on the likelihood of achieving a molecular diagnosis of 2.1. Kim et al. have previously reported a risk ratio of 2.1 in a cohort with retinitis pigmentosa (Kim et al., 2021). However, they also reported that individuals with a positive family history had a significantly earlier age of onset, which we did not observe. The median age of onset among individuals with a positive family history in our cohort was 15 years, compared to 20 years among those without family history, but the difference was not statistically significant (*p* = 0.1, Wilcoxon rank‐sum test). Additionally, the results from the multivariable logistic regression model indicated no significant interaction effects or evidence of multicollinearity. This suggests that age of onset and family history status function as independent predictors of the likelihood of achieving a molecular diagnosis. Consanguinity was also tested for its association with achieving a molecular diagnosis. A trend towards statistical significance was observed (*p* = 0.056). Expanding the sample size or focusing the analysis on individuals with suspected autosomal recessive inheritance may strengthen this association.

Third, we explored the effects of increased genetic testing on a population level. In the Stockholm region, the number of genetic tests per million inhabitants rose 2.1‐fold between the two time periods studied. This increase coincided with the European and Swedish recommendations for genetic testing in all individuals with suspected or clinically diagnosed IRDs in 2021 and 2022 as well as the Swedish approval of voretigene neparvovec‐rzyl (Luxturna) in 2021. The significant rise in testing suggests that, historically, genetic testing may have been limited to individuals with a higher pretest probability for achieving a molecular diagnosis. This is reflected in the clinical predictors age of onset and family history status. After 2021, the median age of onset was increased by 12 years and the proportion with a positive family history was decreased by 12%.

The most important effect of increased genetic testing was a 1.6‐fold increase in the yearly number of diagnosed individuals per million inhabitants. This was accompanied by a decrease in diagnostic yield from 64% to 48%, which is still a high yield compared to other rare diseases (Stranneheim et al., 2021). Although diagnostic yield is an important variable and easy to measure, it should be balanced by a focus on the number of diagnosed individuals. The purpose of genetic testing is to establish the molecular diagnosis for the benefit of the affected individuals and their families. Moreover, a focus on maximizing diagnostic yield could promote a narrow selection of individuals for genetic testing.

The overall diagnostic yield of 55% in our cohort falls at the lower end of the range from 52% to 74% estimated in a recent meta‐analysis (Britten‐Jones et al., 2023). Several factors could be associated with this. First, the increased use of genetic testing over time has resulted in a cohort with older age of onset and where a lower proportion has a positive family history. Second, only one affected individual per family was included. Inclusion of multiple affected individuals from the same family could inflate the diagnostic yield. Third, following the implementation of clinical genome sequencing, our bioinformatic pipeline has not been evaluated on ORF15 in the *RPGR* gene. ORF15 is challenging to sequence and is expected to account for 60% of disease‐causing variants in *RPGR*, the most common associated gene with retinitis pigmentosa (Chiang et al., 2018). Although no variants in ORF15 were detected by genome sequencing in our cohort, two disease‐causing variants have since been identified after the study period. This suggests that some variants in ORF15 are detectable by short‐read genome sequencing. We anticipated that the implementation of clinical genome sequencing would lead to the discovery of more deep‐intronic disease‐causing variants, which have been suggested to account for a significant portion of the missing heritability in IRDs (Schneider et al., 2022; Weisschuh et al., 2020). However, in our cohort of 166 individuals analysed by genome sequencing, only two novel deep‐intronic variants were identified: *NMNAT1* c.‐71G > A (likely pathogenic) and *WDR19* c.1982 + 963G > A (VUS). The low incidence is consistent with findings from a study of 1000 individuals with IRDs analysed by genome sequencing (Weisschuh et al., 2024). These results suggest that bioinformatic pipelines may struggle to detect deep‐intronic variants or that such variants are, in fact, less common than expected. Additionally, functional analysis by RNA‐sequencing to identify deep‐intronic variants that affect splicing is challenging because tissue from the retina is inaccessible and many IRD genes have low or absent expression in blood and skin. Retinal organoids from patient‐derived induced pluripotent stem cells have been shown to have a transcriptome closer to that of the retina (Bronstein et al., 2020). Another tissue that might be suitable for functional analysis of IRD gene is the hair follicle, which has been shown to express full‐length *ABCA4* transcripts (Sciezynska et al., 2020).

In conclusion, this study describes the clinical and genetic findings in a Swedish IRD cohort and identifies age of onset and family history status as significant predictors for achieving a molecular diagnosis. Increased use of genetic testing on a population level substantially increases the number of individuals receiving a molecular diagnosis with a high diagnostic yield compared to other rare diseases.

## Supporting information


**Table S1:** Supporting Information

**Table S2:** Supporting Information

## Data Availability

Non‐sensitive data supporting the findings of this study are available from the corresponding author upon request. Data that compromise patient confidentiality are not publicly accessible.
